# Endovascular treatment of iatrogenic axillary artery pseudoaneurysm under echographic control: A case report

**DOI:** 10.1186/1749-8090-6-78

**Published:** 2011-05-27

**Authors:** Daniela Mazzaccaro, Giovanni Malacrida, Maria T Occhiuto, Silvia Stegher, Domenico G Tealdi, Giovanni Nano

**Affiliations:** 1 University of Milan, Italy. 1st Unit of Vascular Surgery, IRCCS Policlinico San Donato, 20097 San Donato Milanese (MI), Italy

## Abstract

**Aim:**

Brief case report of the treatment of a large axillary artery pseudoaneurysm after a pacemaker using a left brachial cutdown and a retrograde delivery of a covered stent using ultrasound and fluoroscopic guidance. The patient's renal function precluded the use of contrast materials.

**Case Report:**

A 77 years old man presenting with acute renal failure and haemoglobin decrease arrived with an expanding pseudoaneurysm of the left axillary artery from a pacemaker placement. Considering the site of the lesion and patient's comorbidities, under echographic control, a Hemobahn^® ^stent-graft was placed; fluoroscopy assisted manipulation of guidewires and sheaths into the aortic arch. The procedure was successfully ended without any complications. At 8 months the stent graft was still patent.

**Conclusion:**

Ultrasound guidance may represent an alternative for pseudo-aneurysm exclusion without any use of contrast medium, especially in those patient where lesions are easily detectable using ultrasonography and when comorbidities contraindicate aggressive surgical or angiographic approach.

## Introduction

A pseudoaneurysm is a rare but serious complication after pace-maker placement procedures. Because of the risk of expansion and rupture, prompt repair is indicated[1]. Endovascular procedures currently represent a preferred treatment for these lesions, as they are less invasive than surgical approach. Endovascular repair, however, implicates the use of a iodine contrast medium, which may represent a contraindication for patients with a severe renal impairment.

We report here the first case of endovascular exclusion of an axillary artery pseudoaneurysm under ultrasound guidance, without any use of contrast medium.

## Case presentation

A 77 years old man was admitted to our hospital for a sudden pain under his left clavicle, with a large palpable pulsing mass. Two weeks before, he had undergone a pacemaker positioning procedure to manage an arrhythmia.

The patient suffered also from coronary artery disease with stable angina, hypertension and type II diabetes mellitus. On admission the patient was anuric and anaemic; his blood lab-tests showed high level of creatinine (4.2 mg/dl); his haemoglobin was 7.2 g/dl compared to 12.4 g/dl he had before the pacemaker positioning procedure. Moreover, he had a severe respiratory insufficiency and he had progressively developed hypostenia and paresis of his left arm within the last hour.

**Figure 1:**
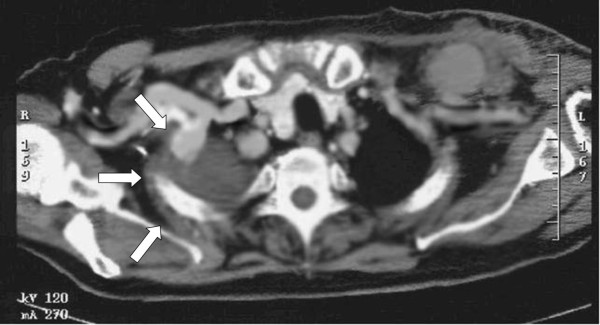
**Preoperative CT-scan**.

A duplex ultrasound was performed, demonstrating a 5.2 cm pseudoaneurysm of the left axillary artery; a thoracic CT-scan without any contrast medium confirmed the lesion along with a large contralateral pleural effusion (Figure 1).

Because of hemodynamic instability and new neurological changes in the left arm, the patient was referred to our unit of vascular surgery for treatment.

Considering the patient's comorbidities and the difficult surgical access we decided that endovascular treatment was indicated. Because of the patient's labile renal function, however, we preferred not to use any iodine contrast medium, so we attempted an endovascular exclusion under echographic guidance. An informed consent was obtained by the Patient.

**Figure 2:**
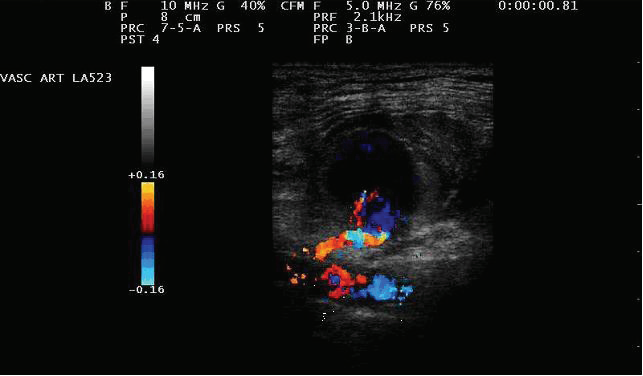
**Duplex scan control**.

MyLab™ 25 X-Vision scan (Esaote S.p.A. Firenze, Italy) with a linear 7-10 MHz probe was used for insonation of axillary, subclavian and vertebral arteries. Preoperative duplex showed the entry point of the lesion and proximal and distal diameter of the axillary artery of 7 and 7.2 mm respectively (Figure 2).

Under loco-regional anesthesia the brachial artery was cannulated in a retrograde fashion with a 5F sheath after surgical exposure. Fluoroscopy was used to assist manipulation of a 0.035-in. hydrophilic guidewire into the aortic arch. Then it was exchanged over a 4F catheter to a 0.020-in. stiff wire (Boston Scientific Meditech) in order to give more support to the entry of the stent-graft. Intraoperative duplex confirmed the proximal and distal diameter of the axillary artery of 7 and 7.2 mm respectively. A 9 × 50 mm Gore Hemobahn^® ^(W.L. Gore Associates, Inc., Flagstaff, AZ, USA) stent-graft was then chosen; this graft is a 0.020" compatible device with a diameter that oversized 20% the vessel diameter. After removal of the 5F sheath, under fluoroscopy, the device was advanced throughout the brachial artery without any sheath due to the inability to advance the proper 9F sheath in such a little vessel. Then, under ultrasound guidance, it was placed across the neck of the pseudoaneurysm and deployed when the correct position was achieved.

No post-dilation was necessary. No intraoperative complications were observed.

**Figure 3:**
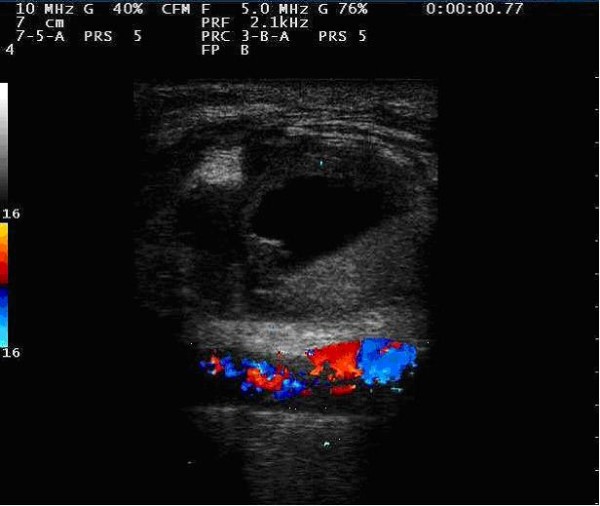
**Post-operative Duplex ultrasound control assessing the complete exclusion of the lesion**.

During the first post-operatory day, the patient received a blood transfusion. His clinical condition gradually improved, and an echographic scan in the third post-operative day showed the complete exclusion of the sac and vessel good patency (Figure 3).

He was discharged five days later with a normal renal function and haemoglobin blood level of 10.4 g/dL.

Eight months later a contrast-enhanced CT-scan control confirmed the complete exclusion of the sac in absence of any endoleaks, and an ultrasound evaluation excluded any flow impairment during upper limb movements.

## Discussion

Iatrogenic axillary artery pseudoaneurysms are uncommon complications of many invasive manoeuvres by transbrachial approach[1]. As reported in literature, the incidence of iatrogenic pseudoaneurysms ranges from 0.1 to 6%[2], but the number of upper limb pseudoaneurysms is even lower (less than 2% of all lesions)[1]. The therapeutic practice in the management of iatrogenic pseudoaneurysms has changed over the last decade. A conventional surgical approach in the axillary area may be associated with many complications, such as major blood loss and potential damage of adjacent neurovascular structures. The surgical inaccessibility of axillary arteries makes endovascular procedure like stent graft placement or thrombin injection particularly attractive [1,3,4].

In our case, the lack of significant published experience with thrombin injections in axillary artery pseudoaneurysms[5], the difficult surgical exposure and the associated patient's comorbidities, especially renal failure, have meant that endovascular repair with a covered stent using sonographic control was the approach of choice.

Considering the anatomy of the involved district, an open surgical approach would have need an anterior thoracotomy above the nipple in the left third or fourth intercostal space[6] with a potential sternotomy to obtain a better exposure and proximal control, but this approach would have worsened the patient's already impaired respiratory function. As an alternative, a supraclavicular incision with a transection of the clavicle would have been required, implying a greater post-operative pain and a longer post-operative course[7].

As for ultrasound-guided thrombin injection, Hirsch et al.[3] presented an approach to the management of catheter-related femoral artery pseudoaneurysms: in the reported algorithm, non-operative intervention such as U.S.-guided compression or thrombin injection are not the option of choice in presence of a symptomatic pseudoaneurysm which is rapidly expanding, causing nerve compression. As there are no clear guidelines about the treatment of axillary district pseudoaneurysms, we referred to this algorithm, so thrombin injection was avoided because of the presence of neurological compression by the hematoma that was progressively enlarging.

Some case reports [8-10] have demonstrated the feasibility of endovascular treatment of an axillary aneurysm until now. In our Centre, 12 endovascular treatment of subclavian-axillary arteries aneurysms have been performed for the last five years, using traditional endovascular methods and with good technical and clinical results. Because of important acute renal failure, in this particular case a less invasive approach was preferred, treating the patient with endovascular technique under ultrasound guidance. Using echography., the apposition of the stent-graft to the vessel wall could likely be assessed as well; to our knowledge, this report is the first case of an endovascular treatment for axillary pseudoaneurysm ultrasound-guided.

The fist decision concerned the access. To avoid the use of any contrast material, we preferred surgical exposure of the brachial artery with a retrograde approach to the lesion. This approach permitted an easy placement of the guide wire in the aortic arch with a very low risk of embolic cerebral events due to manipulation in a very calcified aortic arch, even if it required a surgical cut. Some authors[11] proposed a pre-operatory contrast-MRI evaluation of the aortic arch in order to avoid difficult manoeuvres and large consume of contrast medium during supra-aortic vessels cannulation, above all in case of a bovine conformation; as some past and recent studies[12] reported about the role of gadolinium in triggering renal insufficiency, we preferred, in this particular case, a brachial retrograde approach, which probably was the best option also considering the clinical emergency.

The second issue was about the choice of the stent-graft. In our institution, three stent-graft are always available: Fluency Plus^® ^(Bard Peripheral Vascular Inc, Tempe, Az, USA), Wallgraft^® ^(Boston Scientific, San Francisco, CA, USA) and Hemobahn^® ^(W.L. Gore Associates, Inc., Flagstaff, AZ, USA.). Fluency device did not fit our lesion because of his strong radial force (not recommended for joint positioning) and the presence of flared bare stents (in our experience dangerous for the vessel wall). In order to avoid shortening of the device, a Gore Hemobahn^® ^graft was chosen instead of Wallgraft^®^. The main disadvantage of this stent is in fact shortening, which makes precise placement difficult.

The third issue concerned the follow-up. In this case the normalisation of renal function permitted the execution of a CT-scan with contrast medium, but maybe a simple Duplex scan would have been satisfactory alike. Some authors reported a significant intimal hyperplasia at follow-up, especially in case of repair of traumatic axillary artery pseudoaneurysms[13]; in our case however, placing a covered stent was probably the best therapeutical choice, even if a so highly mobile artery could be prone to neointimal proliferation and stent occlusion.

## Conclusions

Ultrasound guidance may represent an alternative for pseudo-aneurysm exclusion without any use of contrast medium, especially in those patient where lesions are easily detectable using ultrasonography and when comorbidities contraindicate aggressive surgical or angiographic approach.

## Competing interests

The author declares that they have no competing interests.

## Authors' contributions

DM participated in the design of the case report and performed the search in the literature.

GM, MTO, SS, DGT, GN participated in the design and coordination of the report.

All authors read and approved the final manuscript.

## IRB Approval

Our institution approved the report of this case.

## Consent

Written informed consent was obtained from the patient for publication of this case report and accompanying images. A copy of the written consent is available for review by the Editor-in-Chief of this journal.

## References

[B1] Szendro G Golcman L Klimov A Yefim C Johnatan B Avrahami E Yechieli B Yurfest S Arterial false aneurysm and their modern management Isr Med Assoc J 2001 3 1 5 8 11344804

[B2] Görge G Kunz T Kirstein M Non-surgical therapy of iatrogenic false aneurysms Dtsch Med Wochenschr 2003 128 1-2 36 40 12510248 10.1055/s-2003-36329

[B3] Hirsch AT Haskal ZJ Hertzer NR Bakal CW Creager MA Halperin JL Hiratzka LF Murphy WR Olin JW Puschett JB Rosenfield KA Sacks D Stanley JC Taylor LM Jr White CJ White J White RA Antman EM Smith SC Jr Adams CD Anderson JL Faxon DP Fuster V Gibbons RJ Hunt SA Jacobs AK Nishimura R Ornato JP Page RL Riegel B ACC/AHA 2005 Practice Guidelines for the management of patients with peripheral arterial disease (lower extremity, renal, mesenteric, and abdominal aortic): a collaborative report from the American Association for Vascular Surgery/Society for Vascular Surgery, Society for Cardiovascular Angiography and Interventions, Society for Vascular Medicine and Biology, Society of Interventional Radiology, and the ACC/AHA Task Force on Practice Guidelines (Writing Committee to Develop Guidelines for the Management of Patients With Peripheral Arterial Disease): endorsed by the American Association of Cardiovascular and Pulmonary Rehabilitation; National Heart, Lung, and Blood Institute; Society for Vascular Nursing; TransAtlantic Inter-Society Consensus; and Vascular Disease Foundation Circulation 2006 113 11 e463 654 16549646 10.1161/CIRCULATIONAHA.106.174526

[B4] Kumar RM Reddy SS Sharma R Mahajan R Talwar KK Endovascular repair of a traumatic axillary artery pseudoaneurysm Cardiovasc Intervent Radiol 2009 32 3 598 600 10.1007/s00270-009-9543-5 19296160

[B5] Elford J Burrell C Roobottom C Ultrasound guided percutaneous thrombin injection for the treatment of iatrogenic pseudoaneurysms Heart 1999 82 526 527 10490575 10.1136/hrt.82.4.526 PMC1760292

[B6] Rich NM Mattox KL Hirshberg A Vascular Trauma 2004 2 Elsevier Saunders

[B7] Lin PH Koffron AJ Guske PJ Lujan HJ Heilizer TJ Yario RF Tatooles CJ Penetrating injuries of the subclavian artery Am J Surg 2003 185 6 580 4 10.1016/S0002-9610(03)00070-9 12781890

[B8] Marston WA Criado E Mauro MA Transbrachial endovascular exclusion of an axillary artery pseudoaneurysm with PTFE-covered stent J Endovasc Surg 1995 2 172 176 10.1583/1074-6218(1995)002<0172:TEEOAA>2.0.CO;2 9234131

[B9] Sullivan TM Bacharach JM Perl J Gray B Endovascular management of unusual aneurysms of the axillary and subclavian arteries J Endovasc Surg 3 4 389 395 10.1177/152660289600300406 8959496

[B10] Vijayvergiya R Kumar RM Ranjit A Grover A Endovascular management of isolated axillary artery aneurysm Vasc Endovasc Surg 2005 39 2 199 201 10.1177/153857440503900211 15806283

[B11] Ascher E Hingorani AP Marks NA Duplex-assisted internal carotid artery baloon angioplasty and stent placement Perspect Vasc Surg Endovasc Ther 2007 19 1 41 7 10.1177/1531003506298146 17437978

[B12] Solomon GJ Rosen PP Wu E The role of gadolinium in triggering nephrogenic systemic fibrosis/nephrogenic fibrosing dermopathy Arch Pathol Lab Med 2007 131 1515 16 17922586 10.5858/2007-131-1515-TROGIT

[B13] Onal B Ilgit ET Kosar S Akkan K Gümüs T Akpek S Endovascular treatment of peripheral vascular lesions with stent-grafts Diagn Intervent Radiol 2005 11 3 170 174 16206061

